# Bocavirus Infection in Hospitalized Children, South Korea

**DOI:** 10.3201/eid1208.060261

**Published:** 2006-08

**Authors:** Ju-Young Chung, Tae Hee Han, Chang Keun Kim, Sang Woo Kim

**Affiliations:** * Inje University College of Medicine, Seoul, South Korea

**Keywords:** Human bocavirus, South Korea, children, respiratory tract infection, dispatch

## Abstract

This study presents the first evidence of human bocavirus infection in South Korean children. The virus was detected in 27 (8.0%) of 336 tested specimens, including 17 (7.5%) of 225 virus-negative specimens, collected from children with acute lower respiratory tract infection.

Human respiratory syncytial virus (HRSV), human metapneumovirus (HMPV), parainfluenzavirus, coronavirus, and adenovirus are commonly detected viruses in children with acute lower respiratory tract infections (LRTI) (*1**–**5*). Recently, human bocavirus (HBoV) was identified as a cause of human respiratory tract infections in Sweden (*6*). Although HBoV was initially suspected to be an important human respiratory pathogen, the worldwide prevalence and clinical significance of the infection are still unclear. The purpose of this study was to investigate the prevalence of HBoV infection in children hospitalized with acute LRTI in South Korea.

## The Study

A total of 336 specimens were evaluated for the presence of HBoV, including 225 virus-negative specimens (median age of patients = 14 months; range 1–69 months) and 111 virus-positive specimens (median age of patients = 15 months; range 1–83 months). The virus-positive specimens comprised 90 specimens with HRSV, 19 with HMPV, and 2 with adenovirus and were taken from hospitalized children with LRTI at Sanggyepaik Hospital from July 2004 through October 2005. LRTI included the diagnoses of bronchiolitis, bronchitis, pneumonia, and laryngotracheobronchitis. The 336 nasopharyngeal samples were consecutively collected at admission, after informed consent was obtained from the children's parents. Common respiratory viruses (HRSV, adenovirus, influenza A and B, parainfluenza) were detected by direct fluorescent-antibody assay kit (Dako Imagen, Cambridgeshire, UK), after admission. The remaining samples were stored at –70°C for further studies. Viral RNA was extracted from each sample by a QIAamp viral RNA mini kit (Qiagen, Hilden, Germany), and reverse transcription of 0.5 μg of each RNA sample was performed. Reverse transcription–PCR was performed to detect HMPV by using F-gene primers (*7*) and human coronavirus NL-63, by using a 1-a and a 1-b primer, as previously described (*8*).

DNA was extracted from the nasopharyngeal aspirates with a QIAamp DNA Blood Mini Kit (Qiagen GmbH, Hilden, Germany). Two PCR assays were performed for each sample, 1 for the NP1 gene and the other for the NS1 gene, using different primer sets;188F (5´-GACCTCTGTAAGTACTATTAC-3´) and 542R (5´-CTCTGTGTTGACTGAATACA G-3´) for the NP1 gene; and HBoV01.2 (5´-TATGGCCAAGGCAATCGTCCAAG-3´) and HBoV02.2 (5´-GCCGCGTGAACATGAGAAACAGA-3´) for the NS1 gene, as previously described (*6*,*9*). Each cycle comprised predenaturation at 95°C for 3 min and 35 amplification cycles (denaturation at 95°C for 1 min, primer annealing [at 54°C for NP1 gene and 56°C NS1 gene] for 1 min, and extension at 72°C for 1 min). The amplified DNA fragments for the NP1 gene and NS1 gene were 354 bp and 291 bp, respectively. To validate the amplification process and exclude carryover contamination, positive and negative controls were run for each PCR, and positive samples were verified against an independent RNA extraction.

Amplicon was purified by using QIAquick (Qiagen GmbH) and sequenced in both directions with the BigDye Terminator v3.1 Cycle Sequencing kit (Applied Biosystems, Foster City, CA, USA). Sequencing products were resolved with an ABI 3730 XL autoanalyzer (Applied Biosystems). Nucleotides sequences were aligned with BioEdit v7.0 and presented in a topology tree, prepared in MEGA 3.1 (*10*).

## Conclusions

This study presents the first evidence of HBoV infection in Korean children, which suggests that HBoV can infect humans worldwide (*1**,**2*). In this study, HBoV was detected in 27 (8.0%) of 336 tested specimens and in 17 (7.5%) of 225 virus-negative specimens collected from children with acute LRTI. A total of 15 (55.6%) of 27 HBoV-positive specimens were obtained from <1-year-old children, 8 (29.6%) of 27 1- to 2-year-old children, and 4 (14.8%) of 27 3- to 5-year-old children. Other viruses were also detected in 10 (37%) of 27 HBoV-positive specimens; 5 specimens also contained HRSV, 4 had HMPV, and 1 had adenovirus. Although HBoV was detected mostly in the winter in previous studies (*6**,**9*), we detected it throughout the study period.

**Figure 1:**
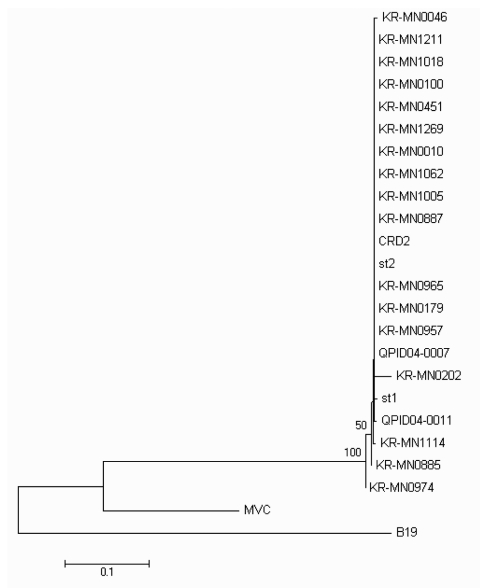
Phylogenetic analysis of Korea (KR-MN), Sweden (st), USA (CRD2), and Queensland (Q) NS1 gene sequences from human bocavirus strains presented on a topology tree prepared in MEGA3.1. Nucleotide alignment of a 245-bp portion of the NS1 gene was prepared by using BioEdit v7.0. The nucleotide distance matrix was generated with Kimura 2-parameter estimation. Nodal confidence values indicate the results of bootstrap resampling (n = 1,000). GenBank accession no. B19 (human erythrovirus B19, DQ408301); MVC (canine minute virus, NC_004442); st1 (HBoV strain st1, DQ000495); st2 (HBoV strain st2, DQ000496); QPID04-0007 (DQ200648); QPID04-0011 (DQ206702); CRD2 (DQ340570).

**Figure 2:**
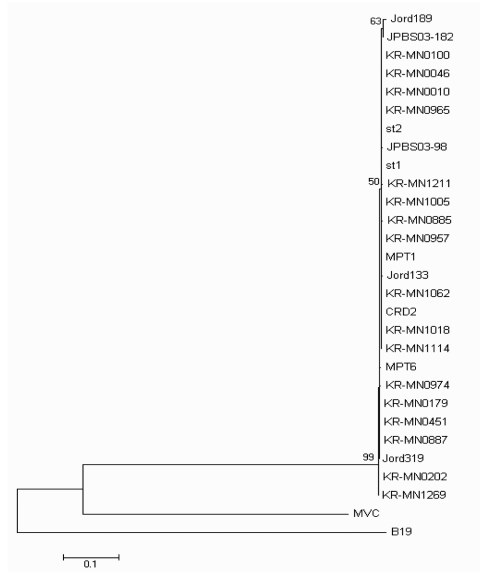
Phylogenetic analysis of Korean (KR-MN), Swedish (st), Jordanian (Jord), French (MP), USA (CRD2), and Japanese (JPBS) NP1 gene sequences from human bocavirus strains presented on a topology tree prepared in MEGA3.1. Nucleotide alignment of a 310-bp portion of the NP1 gene was prepared by using BioEdit v7.0. The nucleotide distance matrix was generated with Kimura 2-parameter estimation. Nodal confidence values indicate the results of bootstrap resampling (n = 1,000). GenBank accession nos. B19 (human erythrovirus B19, DQ408301); MVC (canine minute virus, NC_004442); st1 (HBoV strain st1, DQ000495); st2 (HBoV strain st2, DQ000496); Jord37 (AB243566); Jord133 (AB243567); Jord189 (AB243568); Jord319 (AB243570); JPBS03-98 (DQ296618); JPBS03-182 (DQ296620); JPBS05-52 (DQ296635); MPT1 (AM109958); MPT6 (AM109964); CRD2 (DQ340570).

The medical records of 17 patients with samples positive for only HBoV were retrospectively reviewed. The patients ranged in age from 1 to 37 months of age, and the male-to-female ratio was 2.8:1.The clinical manifestations of HBoV-positive patients were fever (76.4%), cough (76.4%), rhinorrhea (23.5%), gastrointestinal symptoms (11.7%), and rashes (5.8%). Direct sequencing of PCR products of the NS1 gene and NP1 genes showed that most strains had the same sequences (Figure 1 and Figure 2). The prevalence of HBoV in this study was higher than that in previous studies (3.14%–5.7%). This variation may be due to differences in the characteristics of the study populations and collection time of respiratory specimens (*6**,**9**,**11*). In our study, the positive rates of HBoV in the nasopharyngeal aspirates of patients with acute LRTI were relatively high. However, the clinical role of HBoV is still unclear because of a high frequency of co-infection (37%), a finding similar to those of previous studies (17.6%–55.6%) (*6**,**9*). In a future study, additional microbial testing for rhinovirus and coronaviruses, which are known to cause LRTIs, is needed to evaluate the precise rate of detection of >1 virus (*12**,**13*).

In conclusion, we confirmed HBoV infection in hospitalized children with acute LRTI in South Korea. Further prospective population-based studies are needed to confirm the role of HBoV in LRTI in children.
